# Side-by-side evaluation of two Crimean-Congo hemorrhagic fever virus isolates in IFNAR^−/−^ mice

**DOI:** 10.1038/s44298-026-00216-2

**Published:** 2026-07-31

**Authors:** Cornelius Rohde, Anke-Dorothee Werner, Michelle Gellhorn Serra, Petra Emmerich, Nataša Knap, Markus Eickmann, Stephan Becker, Verena Krähling, Alexandra Kupke, BSL4 Animal Facility Team, Marcel Benz, Martina Huxol, Lennart Kämper, Michael Klüver, Lars Meier, Pauline Neubecker

**Affiliations:** 1 Philipps-Universität Marburg, Marburg, Fachbereich Medizin, Institut für Virologie, Marburg, Germany; 2 German Center for Infection Research (DZIF) Partner Site Gießen-Marburg-Langen, Marburg, Germany; 3 Department of Virology, Bernhard Nocht Institute of Tropical Medicine, Hamburg, Germany; 4 Department of Tropical Medicine and Infectious Diseases, Center of Internal Medicine, University Medicine Rostock, Rostock, Germany; 5 Institute of Microbiology and Immunology, Faculty of Medicine, University of Ljubljana, Ljubljana, Slovenia

**Keywords:** Diseases, Immunology, Microbiology

## Abstract

Crimean-Congo hemorrhagic fever virus (CCHFV) is the causative agent of a severe hemorrhagic fever in humans, associated with case fatality rates up to 40%. Due to the lack of approved vaccines or specific antiviral treatments, CCHFV is classified as a biosafety level 4 (BSL4) pathogen in most countries and designated a priority pathogen by the World Health Organization. To facilitate the preclinical assessment of medical countermeasures, we have established a murine model using C57BL/6J IFNAR^−/−^ mice, which lack the IFNα/β receptor, infected with the phylogenetically distinct CCHFV strains Afghanistan09-2990 (Afg09) and Kosovo Hoti (Hoti). Infection with both CCHFV strains in IFNAR^−/−^ mice resulted in significant weight loss, with Afg09 infection leading to more severe clinical disease. Quantitative analysis of viral RNA revealed widespread viral dissemination across multiple organs. Detection of infectious virus varied by organ and strain. These results independently validate previously reported disease phenotypes while providing new insight into strain-specific differences in CCHFV pathogenesis in IFNAR^−/−^ mice. Thereby, these CCHFV models represent valuable tools for the evaluation of antiviral therapeutics and vaccine candidates, enabling the investigation of cross-lineage protection against genetically diverse CCHFV isolates.

## Introduction

Crimean-Congo hemorrhagic fever virus (CCHFV) is mainly transmitted by tick bites to humans and can cause severe hemorrhagic fever, with 1000–2000 fatalities per year worldwide^1^. Due to the widespread distribution of the main vector (ticks of the genus *Hyalomma*), CCHFV transmission has been reported in Europe, Africa and Asia. This broad geographic spread is reflected in classification of the virus into five distinct genetic lineages (I–V)^2^. In the absence of approved antiviral therapeutics or vaccines, CCHFV is listed as a priority pathogen for research by the World Health Organization (WHO), capable of causing a Public Health Emergencies of International Concern (PHEIC)^3,4^.

Preclinical animal models are still required to investigate the efficacy of therapeutics and vaccines against emerging viruses such as CCHFV. For this purpose, transgenic rodent models with defects in their type I interferon response are widely used. In contrast to wild-type mice, those animals develop a lethal disease after CCHFV infection. IFNAR^−/−^ mice, which lack the type I interferon receptor, or STAT1^−/−^ mice, which lack the signal transducer and activator of transcription 1, a key transcription factor downstream of type I, II, and III interferon signaling, are suitable transgenic mouse lines for efficacy studies^5–7^. In addition, wild-type mice whose interferon response is downregulated with monoclonal antibodies succumb to CCHFV infection^3,7–9^. To prepare for studies that aim at evaluating cross-protection capacities of future CCHFV vaccines, it was interesting to evaluate the disease development of phylogenetically different CCHFV strains in the same mouse model.

In the present study, we infected C57BL/6J IFNAR^−/−^ mice with CCHFV Afghanistan09-2990 (Afg09, Lineage IV, Asia) or CCHFV Kosovo Hoti (Hoti, Lineage V, Europe) and compared infection characteristics. Both isolates have previously been evaluated in IFNAR knockout and antibody-mediated IFNAR knockdown mouse models, revealing differences in the course of infection between Afg09 and Hoti^8,10–13^. Building on the findings of Golden et al.^13^, the present study directly compared these two CCHFV isolates to assess disease progression and viral replication kinetics, while also providing new insights at the level of individual animals.

## Results

### Clinical course of CCHFV Afg09 and Hoti infection

**Fig. 1:**
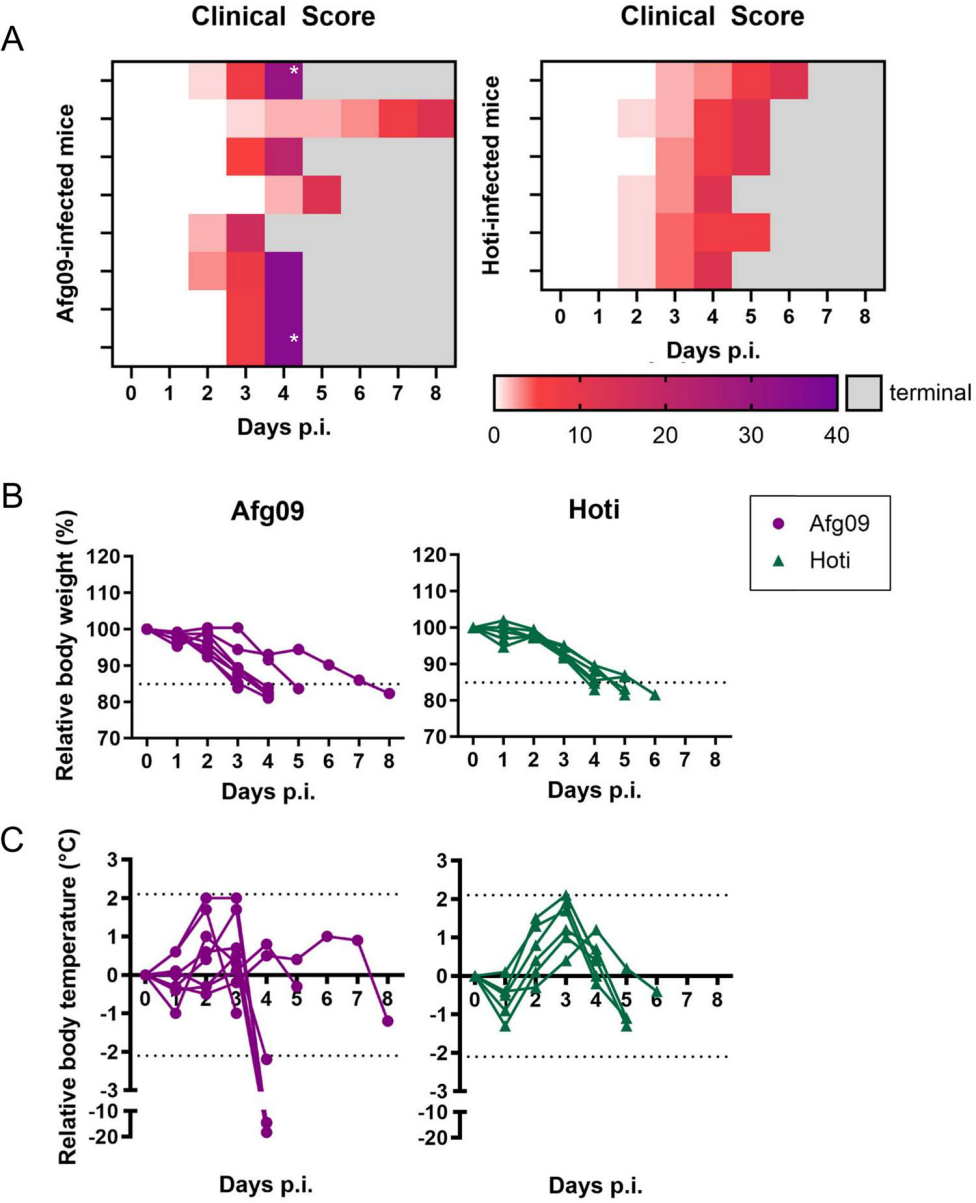
Clinical course of CCHFV Afg09 and Hoti-infected IFNAR^−/−^ mice. IFNAR^−/−^ mice were infected with either 100 TCID_50_ CCHFV Afg09 (purple, n = 8) or Hoti (green, *n* = 6). The clinical score **(A)**, summarizing appearance, behavior, body weight (**B**), and body temperature (**C**), was monitored daily. After reaching the clinical endpoint (score of ≥10 or ≥6 on two consecutive days), the mice were euthanized or found dead (terminal, gray). Asterisk indicates mice that have died. Dotted lines mark the clinical endpoints.

In this study, we established a mouse model for CCHFV Afg09 and Hoti, respectively, and analyzed them side-by-side to compare the pathogenesis of both CCHFV isolates. For this purpose, C57BL/6J IFNAR^−/−^ mice were infected intraperitoneally (i.p.) with 100 TCID_50_ of the Afg09 or Hoti isolate. Equal numbers of female and male mice (Afg09: 4 males and 4 females; Hoti: 3 males and 3 females) were used to take into account possible sex-specific effects on the clinical outcome. Body weight, body temperature as well as spontaneous behavior and body condition were assessed at least once daily, to determine a clinical score. A score of up to 10 could be achieved in each of these categories. These scores were then added together, resulting in a maximum possible score of 40. Mice with a clinical score of ≥10/40 or if a clinical score of ≥6/40 was observed on two consecutive days were euthanized. The course of the Afg09-infection in two mice was extremely rapid, resulting in spontaneous deaths on day 4 p.i. (2/8, 25%). They were included in the scoring for that day and assigned the maximum score for the affected clinical parameters before being recorded as deceased (Fig. 1A and Figs. S1, 2). All other mice infected with CCHFV Afg09 reached the clinical endpoint (6/8, 75%) between days 5-8 post infection (p.i.) (Figs. 1A and Figs. S1, 2). The average clinical score after Afg09 infection of 24.6 was reached because of weight loss (Fig. 1B), altered behavior and appearance, and in some cases severely reduced body temperature (Figs. 1C and Figs. S1, 2). All mice infected with CCHFV Hoti reached the humane clinical endpoint (6/6, 100%) at days 4–6 p.i. (Figs. 1A and Figs. S1, 2) with an average clinical score of 11.2, which again correlated with loss of body weight (Fig. 1B and Fig. S1, 2), altered behavior, and appearance (Figs. S1, 2). After infection with both CCHFV isolates, an increase in body temperature (average peak increase of body temperature: Afg09 1.18 °C, Hoti 1.28 °C) was observed 1–2 days prior to reaching the clinical endpoint (Fig. S2). At the clinical endpoint, body temperature declined again, dropping well below the physiological range in some Afg09-infected mice (Fig. 1C and Figs. S1, 2), serving as an additional indicator of disease progression.

### Distinct viral dissemination of CCHFV Afg09 and Hoti

**Fig. 2:**
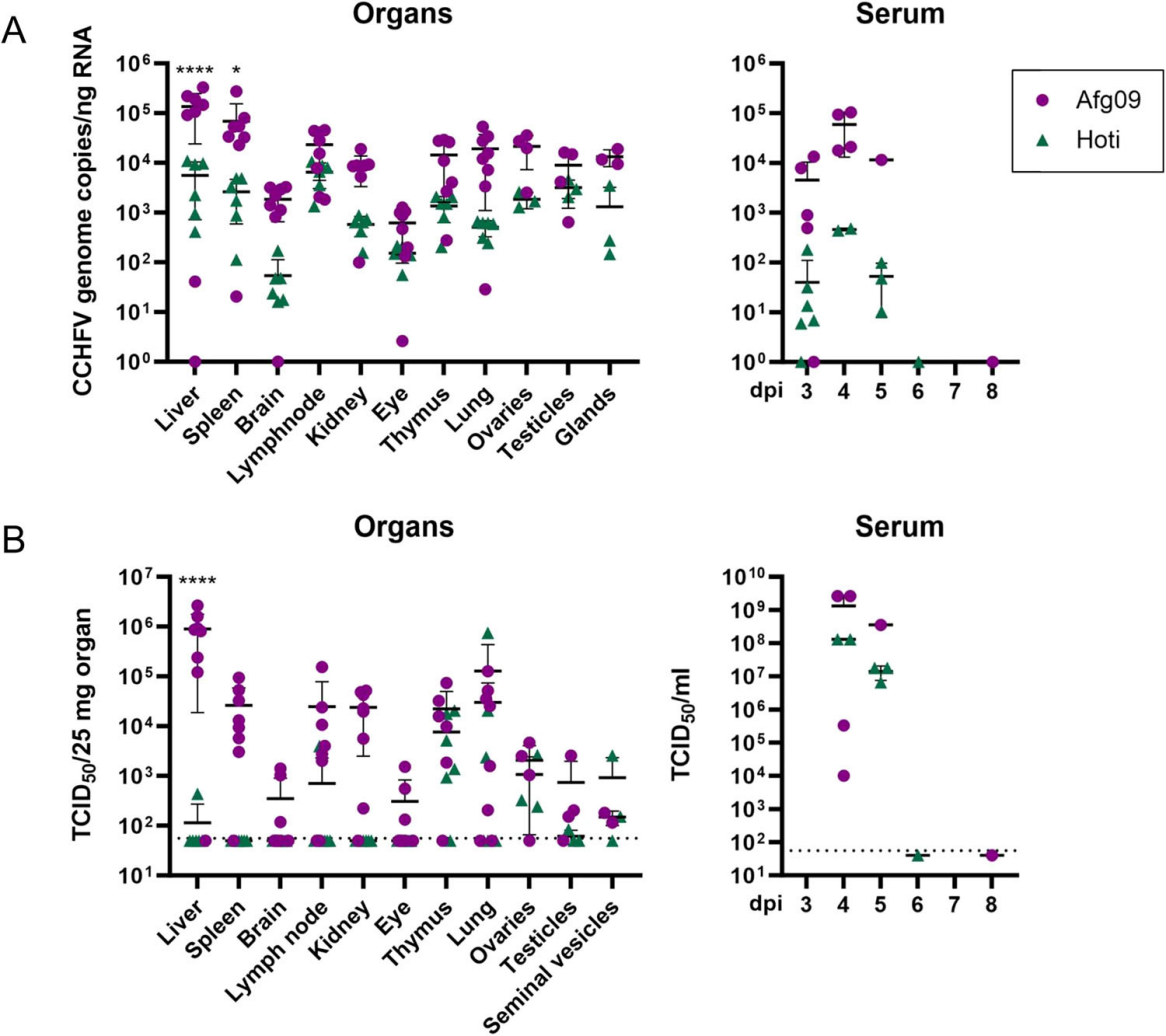
Detection of CCHFV in IFNAR^−/−^ mice infected with Afg09 or Hoti. IFNAR^−/−^ mice were infected with either 100 TCID_50_ CCHFV Afg09 (purple, *n* = 8) or Hoti (green, *n* = 6). **A** CCHFV genome copy was measured by RT-qPCR in organs and serum samples (day 3 and final) of individual mice. **B** Infectious CCHFV was determined by TCID_50_ assay in organs and final serum samples of individual mice. Each data point represents a sample from an individual animal, data are shown as the means ± SD. Datasets were analyzed using Šídák’s multiple comparisons test (**A**) or Tukey’s multiple comparisons test (**B**). Asterisks indicate statistical significance as detailed between CCHFV Afg09 and Kosovo Hoti group: ∗*p* ≤ 0.05; ∗∗∗∗*p* < 0.0001.

During the study, blood was taken from all mice on day 3 p.i. and final blood samples were collected from anaesthetized mice before they were euthanized and organs harvested. To detect CCHFV RNA in organs and serum, qRT-PCR analysis was performed. Virus-specific RNA was detected in serum at day 3 p.i. and at the clinical endpoint, as well as in spleen, liver, brain, lymph nodes, kidney, eye, thymus, lung, ovaries, testes, and seminal vesicles (Fig. 2A and Fig. S2). The amount of infectious CCHFV in organs and final serum samples was determined by TCID_50_ assays. For Afg09, infectious virus was detectable in almost all organs and sera until day 5 p.i. for the majority of mice. Viral titers differed among organs, with the highest viral load detected in the liver and the lowest in the seminal vesicles. Low titers of infectious viruses were detected in individual animals in the brain and the eyes. For Hoti, infectious virus could only be detected in final sera, thymus, lung and ovaries or testicles (Fig. 2B).

**Fig. 3:**
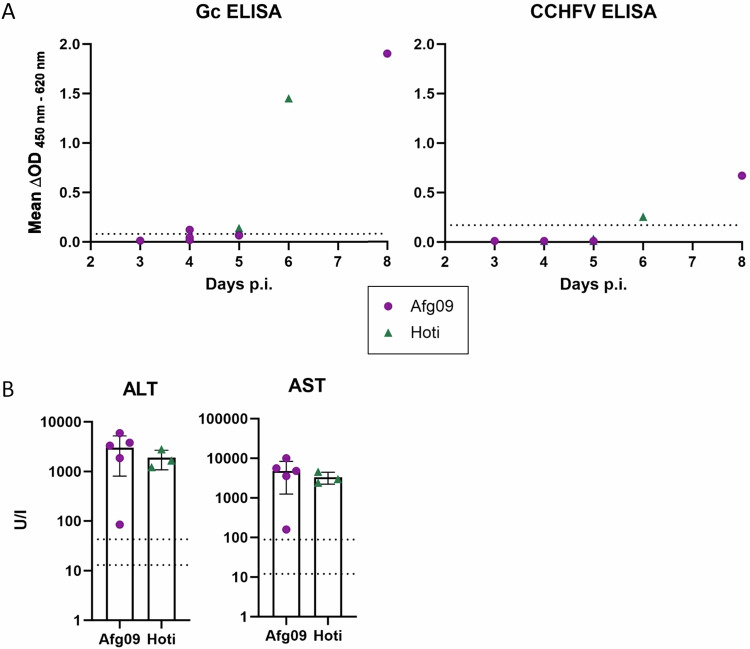
Serology of CCHFV Afg09 and Hoti-infected IFNAR^−/−^ mice. IFNAR^−/−^ mice were infected with either 100 TCID_50_ CCHFV Afg09 (purple) or Hoti (green). **A** Final sera (Afg09 *n* = 6, Hoti *n* = 6) were analyzed with two virus-specific ELISAs using either inactivated CCHFV, measuring NP-specific antibodies, or the major glycoprotein Gc for coating. Monoclonal antibodies detecting Gc or N protein were used as controls (not shown). Dotted lines indicate the respective cut-off. **B** Liver enzymes alanine aminotransferase (ALT) and aspartate aminotransferase (AST) were analyzed in final serum samples (Afg09 *n* = 5, Hoti *n* = 3). Dotted lines indicate the physiological range of healthy C57BL/6 mice^19^.

The humoral immune response (IgG) against CCHFV was tested in clinical endpoint sera using a CCHFV Afg09-specific whole virus ELISA, detecting mainly NP-specific antibodies, and a CCHFV glycoprotein Gc-specific ELISA. Sera from six infected animals per group was available for analysis (Fig. S2). Gc-specific antibodies were first detected at day 4 p.i. in a mouse infected with CCHFV Afg09. Among the four animals that reached the clinical endpoint at day 5 p.i., three mice infected with the Hoti exhibited Gc-specific antibodies in their sera, whereas the Afg09-infected animal did not. Of the two animals that survived beyond day 5, one infected with Hoti (surviving until day 6) and one with Afg09 (surviving until day 8), both developed a broader humoral immune response, with sera containing antibodies specific for both Gc and whole CCHFV antigens Fig. 3A and Fig. S2).

Final serum samples were analyzed for the liver enzymes alanine aminotransferase (ALT) and aspartate aminotransferase (AST) as a sign of hepatocellular damage. Sera from five Afg09- infected (collection time point: 3; 4; 4; 5 or 8 days p.i.) and three Hoti-infected (collection time point: 4; 4 or 5 days p.i.) animals were available for analysis (Fig. S2). This showed that ALT and AST values were well above the physiological reference range either after Afg09 or after Hoti infection (Fig. 3B and Fig. S2).

**Fig. 4:**
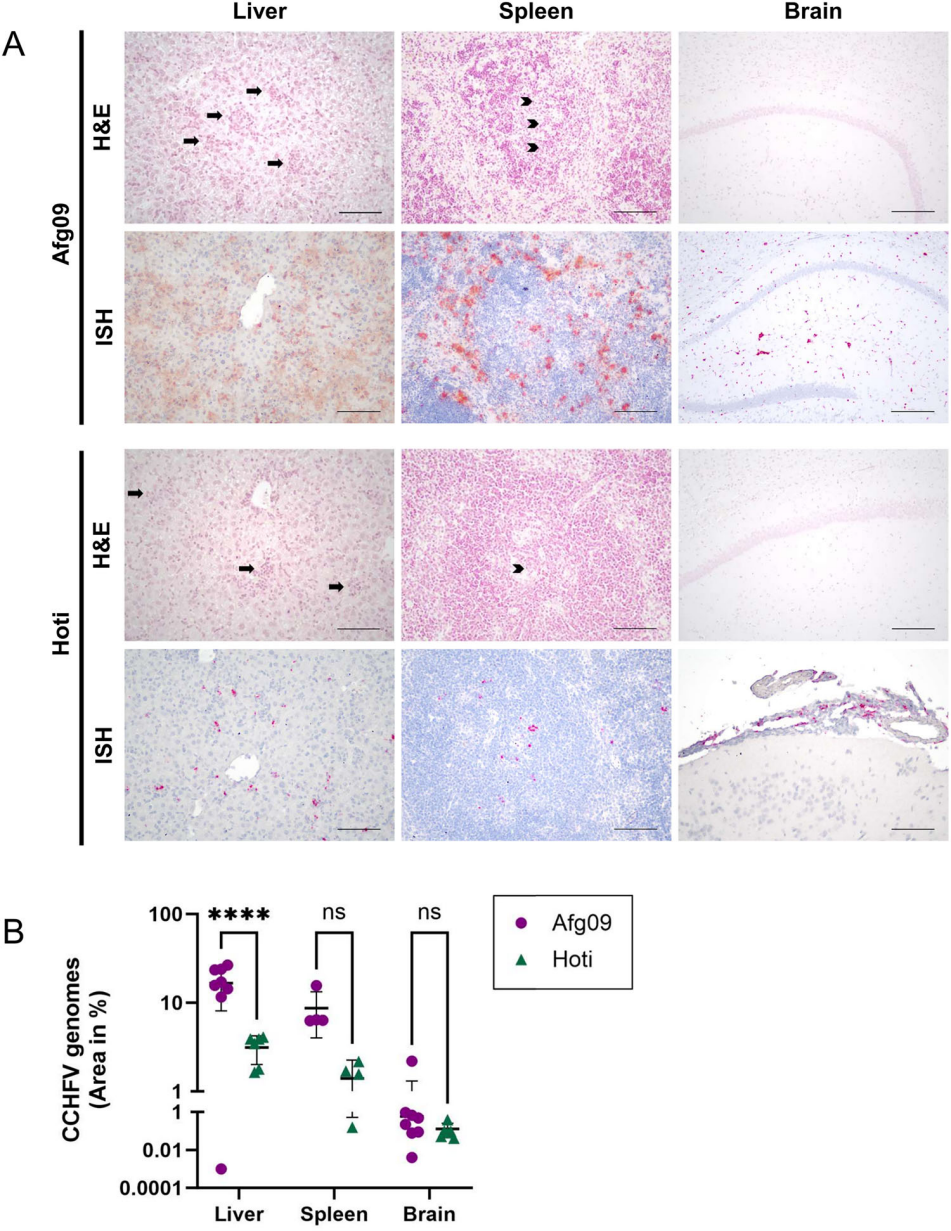
Pathology of CCHFV Afg09 and Hoti-infected IFNAR^−/−^ mice. Liver, spleen and brain of CCHFV Afg09 (purple) or Hoti-infected (green) IFNAR^−/−^ mice were pathologically examined post-mortem. **A** Shown are exemplary images of organ histopathology, H&E (hematoxylin and eosin) staining; ISH (in situ hybridization). Arrows indicate multifocal liver necrosis with loss of tissue architecture and lymphohistiocytic infiltrates. Arrowheads indicate apoptosis in germinal follicle centers. Scale bars: 100 µm. **B** Quantification of ISH indicating percentage area of CCHFV-positive organ sections. Each data point represents a sample from an individual animal, and horizontal lines represent the means ± SD. Datasets were analyzed using Šídák’s multiple comparisons test. Asterisks indicate statistical significance as detailed between CCHFV Afg09 and Kosovo Hoti group: ∗∗∗∗*p* < 0.0001; ns = not significant.

Histopathological analysis of all infected mice (Afg09: *n* = 8; Hoti: *n* = 6) was performed. Hematoxylin and eosin (H&E) stained slides revealed for both CCHFV isolates multifocal liver necrosis and lymphohistiocytic infiltrates (Fig. 4A, arrows). In Afg09-infected mice, these necroses were generally more widespread and occasionally resulted in the complete loss of the tissue architecture. Spleen histopathology indicated apoptosis in germinal follicle centers after CCHFV Afg09 infection and secondary follicles with only a few apoptotic lymphocytes after CCHFV Hoti infection (Fig. 4A, arrowheads). Untreated controls did not reveal histopathological changes (Fig. S3) Interestingly, nearly all organs examined, including the brain with meninges, revealed in situ hybridization (ISH) staining without widespread and marked tissue damage (Fig. 4A). Quantification revealed that CCHFV genomes were detectable by ISH in 17% of the liver area in mice infected with CCHFV Afg09, compared to 3% in mice infected with CCHFV Hoti. In the spleen, viral genomes were detectable in approximately 9% of the tissue area in Afg09-infected mice, whereas only about 1% of the spleen area was CCHFV-positive in mice infected with Hoti. Similarly, in the brain, CCHFV RNA was present in approximately 0.6% of the tissue area following Afg09 infection, compared to 0.1% in Hoti-infected animals (Fig. 4B and Fig. S2). Altogether, ISH revealed a markedly higher abundance of viral RNA in tissues from mice infected with Afg09 compared to those infected with Hoti.

When examining the individual data of all CCHFV-infected mice (Fig. S1A and Fig. S2), it became evident that all animals experienced severe weight loss irrespective of the virus isolate (14/14). In most Afg09-infected mice, the clinical endpoint was further characterized by a marked reduction in body temperature (5/8; Fig. S1A and Fig. S2) and pronounced alterations in spontaneous behavior (6/8; Fig. S1A). The viral load correlated with survival time: the earlier the animals succumbed to infection or were euthanized, the higher the levels of CCHFV genomes detected in serum, liver, and brain (Fig. S2). Conversely, in the two animals in which no viral genomes were detectable in terminal serum samples, elevated levels of Gc-specific IgG antibodies were observed (Fig. S2). Although ALT and AST levels exceeded the physiological range in all tested infected mice (Fig. 3 and Fig. S2), the lowest enzyme values were recorded in the mouse that survived the longest (8 days p.i.). In conjunction with the virological and serological findings (Fig. S2), this suggests that this animal had at least partially overcome the acute phase of infection. Together, these observations indicate a consistent pattern of severe systemic disease across both isolates, with individual variation in viral clearance and immune response likely influencing disease outcome.

## Discussion

In the present study, we established a CCHFV model in C57BL/6J IFNAR^−/−^ mice for CCHFV Afg09 and Hoti. Infections with both viruses took a severe clinical course, with the Afg09 being more fulminant. Both viral isolates led to a clear, temporary increase in body temperature, that was measured 1–2 days before the clinical endpoint was reached. Fever is also one of the pre-hemorrhagic symptoms of CCHF in humans, as reviewed elsewhere^14^. However, comparing the clinical course between the Afg09 and Hoti infection, clear differences were detected. Afg09-infected animals reached humane endpoint criteria between day 4 and 8 characterized by severe weight loss (>15%), reduced spontaneous behavior and altered appearance as well as a preterminal drop in body temperature. Two of the eight animals succumbed to the infection during the course of the experiment between days 3 and 4. This is also reflected in the rapid and dramatic worsening average clinical score of 24.6 points at the time of euthanasia. These findings are consistent with other studies in which the Afg09 strain was lethal in IFNAR^−/−^ mice or when the interferon activity of wild-type mice was counteracted by mouse-specific anti-IFNAR1 monoclonal antibodies^12,13,15^. Hoti-infected mice reached the humane endpoint criteria on day 4–6 mainly because of a severe weight loss (>15%), with few other clinical signs. The final average clinical score was 11.2 points. In other studies using C57BL/6 IFNAR^−/−^ mice, Hoti-infected mice reached the endpoint 8 days after i.p. infection^16^. The different criteria for the humane endpoint, >15% weight loss in our study and >25% in ref. ^16^, may explain the difference in survival time. Notably, in a study by Hawman et al. subcutaneous infection with the CCHFV Hoti isolate even failed to result in a lethal outcome in the majority of infected IFNAR^−/−^ mice^11^. Consistent with these findings, mice in which the type I interferon response was transiently blocked by using mouse-specific anti-IFNAR1 monoclonal antibodies also survived infection with the CCHFV Hoti isolate^13^. This model has therefore been considered suitable for studying recovery following severe CCHFV disease. As humane endpoint criteria are defined individually by each laboratory in agreement with institutional animal welfare officers and local regulatory authorities, outcomes across animal models may vary accordingly. Nonetheless, a weight loss exceeding 15% is generally recognized as a severe clinical burden.

CCHFV infection in humans causes a systemic disease that affects multiple organs including the brain and is frequently associated with a fatal outcome^14^. In the present animal model, post-mortem examinations demonstrated that CCHFV genomes could be detected in all organs and sera examined, with markedly higher viral RNA levels in mice infected with Afg09 compared to those infected with Hoti. Infectious virus was detected in almost all organs tested and in the serum of Afg09-infected animals. Notably, the presence of virus in the brain and eyes suggests a breach of the blood-brain and blood-ocular barriers, allowing viral entry into immune-privileged sites. In Hoti-infected mice, infectious virus was detected primarily in serum, thymus, lung and ovaries.

Previous studies had demonstrated infectious Afg09 in spleen, kidney, liver, heart, lung and brain of infected mice^12^. Our comprehensive analysis further identified viral presence in additional tissues, including thymus, eyes, mesenteric lymph nodes and the reproductive tract of both male and female mice. For Hoti, earlier investigations reported viral genomes in serum, liver, spleen, lymph nodes, lungs, brain, eye and kidneys^11,16^. In the present study, we also detected viral RNA—and, in some cases, infectious virus—in the reproductive tract of both sexes. The relatively low levels of infectious virus in most organs of Hoti-infected mice support the hypothesis that the mice are, in principle, capable of clearing the infection after a few days. This interpretation aligns with earlier findings in which mice infected with Hoti exhibited signs of recovery beginning on day 4 p.i.^11,13^. The detection of CCHFV-specific IgG antibodies in the sera from day 4 p.i. onwards further supports the activation of an adaptive immune response. Animals that remained in the study until day 6 or 8 p.i. exhibited both Gc-specific antibodies and also antibodies that react in the whole CCHFV ELISA. Concurrently, neither CCHFV genomes nor infectious virus was detectable in the sera of these animals. Furthermore, viral RNA levels and infectious viral titers in their organs were markedly lower compared to animals that had to be euthanized at earlier time points. Importantly, the detection of virus in the reproductive tract organs of female and male mice after infection with both CCHFV isolates suggests that CCHFV could also be transmitted sexually. Viral detection in the reproductive tracts of IFNAR^−/−^ mice has also been reported for other CCHFV strains^17^. This is consistent with individual human cases that provide evidence for sexual transmission in both male-to-female and female-to-male directions^18^.

Organ damage resulting from infection with the CCHFV isolates Afg09 and Hoti was further characterized using blood chemistry and histopathological examinations. Blood chemistry analyses revealed significant elevations of liver enzymes (ALT and AST) after infection with both CCHFV isolates compared to the reference range of uninfected C57BL/6 mice^19^, indicating substantial liver damage following infection. Notably, enzyme levels were slightly higher in mice infected with Afg09 compared to those infected with Hoti. These findings align with previous studies suggesting comparable liver damage induced by both virus isolates^10^.

Histopathological analysis confirmed liver damage consistent with serological findings. However, liver and spleen damage were less pronounced in Hoti-infected animals compared to those infected with Afg09. Brain abnormalities were observed only sporadically; however, viral RNA was detectable by ISH in all three analyzed organs. Generally, ISH staining in the brain was weaker in Hoti-infected mice compared to Afg09-infected animals. The severity of tissue damage in individual organs tended to correlate positively with the detected viral load.

A previous study by Golden et al.^13^ also investigated liver pathology in mice infected with these two CCHFV isolates, reporting comparable histopathological findings between isolates at day 4 p.i., consistent with our observations. In contrast to our in situ hybridization results, however, Golden and colleagues found similar viral RNA levels in the liver for both isolates at day 4. This discrepancy partially arises from differences in the timing of analyses; while Golden et al. euthanized animals at a fixed time point (day 4), animals in our study reached the clinical endpoint variably between days 3 and 8 p.i.. Our study, therefore, includes mice in which a detectable humoral immune response emerged from day 5 p.i. onward, coinciding with a subsequent reduction in viral load. This reduction is also demonstrated by Golden et al. by the fact that a significant decline in viral RNA by day 10 following Hoti infection, suggesting active viral clearance from day 4 onwards^13^. However, even when restricting the analysis to mice euthanized on day 4 p.i. (Afg09: *n* = 5; Hoti: *n* = 2), distinct differences between the two CCHFV isolates persist with respect to the levels of viral RNA detected in liver tissue by in situ hybridization.

CCHFV poses a global health threat due to its widespread distribution and genetic diversity, which has resulted in the emergence of at least five distinct viral lineages. To ensure the development of broadly effective therapeutics and vaccines, it is critical to evaluate their efficacy across these diverse strains. To address this, we established side-by-side a CCHFV mouse model using the Afg09 and Hoti isolates, representing the Asian and European lineages, respectively. Comparative analysis revealed that both isolates cause lethal disease in IFNAR^−/−^ mice, although they differ in the severity and progression of illness. Our findings independently confirm previously reported disease characteristics in the IFNAR^−/−^mouse model while extending the field’s understanding of strain-dependent variation in CCHFV pathogenesis. Together, these data support the robustness and reproducibility of this model across distinct studies and reinforce its value for comparative pathogenesis studies and preclinical evaluation of medical countermeasures.

## Methods

### Cells and viruses

Vero C1008 cells (ATCC CRL-1586) were cultured as described elsewhere^20^. CCHFV Afghanistan09-2990 (GenBank: HM452307.1, HM452306.1, and HM452305.1; passage +1 upon receipt) and CCHFV Kosovo Hoti (GenBank: DQ133507, EU037902, and EU044832; passage +1 upon receipt) genomes were confirmed by next-generation sequencing. Comparison of the consensus coding sequences with the respective GenBank reference sequences identified a limited number of nucleotide substitutions. In the Hoti isolate, a synonymous mutation was detected in the M segment (EU037902; G4973A), while the S segment (EU044832) contained two nonsynonymous substitutions (A3466G, Tyr→Cys; G5194A, Ser→Asn). In the Afghanistan09-2990 isolate, one nonsynonymous substitution was identified in the L segment (HM452305; C426T, Ser→Phe) and one in the S segment (HM452307; T7385A, Phe→Leu). All cell cultures and virus stocks were routinely tested and confirmed to be free of mycoplasma contamination prior to use in the experiments. All experiments with CCHFV were carried out in the BSL4 laboratory of Marburg University, Germany.

### Animal experiments

Male and female C57BL/6J IFNAR^−/−^ mice (12–16 weeks old) carrying a knockout (KO) of the interferon alpha/beta receptor (IFNAR) were chosen as a model because they are susceptible to infection with CCHFV^3^. Mice were provided by U. Kalinke (Twincore, Hanover, Germany) and were bred in-house. All experiments and protocols were approved by the local authorities (animal welfare committees; Marburg: Regierungspräsidium Gießen AZ V54 –19 c 20 15 h 01 MR 20/7 Nr. G 87/2022) and conducted according to the recommendations of Federation of European Laboratory Animal Science Associations (FELASA) and the Society for Laboratory Animal Science (GV-SOLAS) and were in compliance with the German animal welfare act as well as the directive 2010/63/EU. Tissue and blood samples from healthy, non-infected control mice were available from previous studies.

Two weeks prior to the start of the experiment, mice were assigned to the experimental groups (2-3 animals per sex) and housed in isocages (Tecniplast, Buguggiate, Italy) under SPF conditions according to FELASA recommendations. One week before the experiment, they were tagged under brief isoflurane anesthesia (CP-Pharma, Burgdorf, Germany) with an ear mark and implanted subcutaneously in the dorsal neck region with a transponder (PTT-300, Plexx BV, Elst, Netherlands) for body temperature measurement. At the start of the experiment, mice were infected intraperitoneally (i.p.) with 100 TCID_50_ CCHFV under brief isoflurane anesthesia. In addition to their normal diet, all animals were given access to a high-calorie energy gel after infection. The mice were monitored daily for weight, body temperature, general condition and spontaneous behavior, with each category receiving a maximum score of 10 (total categorical score range: 0–40). Within each category, a low, moderate, or high burden was assigned a score of 1, 5, or 10 points, respectively, while the absence of clinical abnormalities was scored as 0. The detailed criteria for scoring can be found in Fig. S1C. The sum of the four scores determined the clinical score. When a clinical score of ≥10, or ≥6 on two consecutive days, was reached, mice were euthanized by cervical dislocation under isoflurane anesthesia. This clinical scoring scheme is used as a basis for a standardized scoring system routinely used for all mouse infection studies of the BSL4 Animal Facility. Two out of eight Afg09-infected mice succumbed between days 3 and 4 despite daily monitoring and additional video surveillance before reaching the pre-defined humane endpoint criteria. Consequently, an additional clinical examination was incorporated into subsequent studies on day 3 p.i. in the late afternoon. On days 3 or when the humane endpoint was reached, blood was drawn under brief isoflurane anesthesia by puncture of the facial vein (Microvette® CB 300 Serum CAT, Sarstedt, Nümbrecht, Germany). All subsequent tests (PCR, ELISAs and blood chemistry) were performed using serum.

### Quantitative RT-PCR analysis of virus load in mouse tissue samples

Tissue and serum samples from mice were processed as described elsewhere^21^. RT-qPCR was carried out using Altona diagnostics (Hamburg, Germany) RealStar® CCHFV RT-PCR Kit 1.0 RUO (Cat. No. 181003) according to manufacturer’s instructions on a qTOWER³ (Analytik Jena, Germany).

### Virus titration by TCID_50_ assay

To determine infectious virus in cell culture supernatants or organ and serum samples, 10^4^ Vero C1008 cells were seeded in 96-well plates. The following day, cells were inoculated with fivefold serial dilutions of supernatants from either infected cells, serum samples or organ homogenates. At 6 days p.i. cytopathic effect (CPE) was analyzed via microscopy and TCID_50_/ml values were calculated according to Spearman and Kerber.

### Histopathological examination

Organs were collected after the humane endpoint was reached and processed for histological analysis as described elsewhere^22^. To stain CCHFV RNA, either the Afg09-specific probe (RNAscope Probe - V-CCHFV-M-CPG-sense, Cat. No. 494801, Bio-Techne, Minneapolis, USA) or the Kosovo Hoti-specific probe (RNAscope Probe - V-CCHFV-M-env-sense, Cat. No. 497341, Bio-Techne, Minneapolis, USA) was used, which both target the antisense (vRNA) strand. Infected areas positive for CCHFV viral RNA by ISH were quantified by determining the relative area of tissue staining positive for viral RNA by automated image analysis of the whole section. Whole slides were scanned using the Hamamatsu NanoZoomer system. The images were then converted to 8-bit images using ImageJ and the threshold was adjusted to detect only positive areas. The measurements were set to determine ‘area’ and “area fraction”.

### CCHFV Gc-specific ELISA (IgG)

The CCHFV Gc ELISA was performed in the same way as our SARS-CoV-2 spike protein (S1) ELISA (IgG)^23^. Deviations from the protocol were the following. Microtiter plates were coated with CCHFV glycoprotein (Gc) protein (REC31696, The Native Antigen Company, Oxford, United Kingdom) diluted to 0.5 µg/ml in phosphate-buffered saline (PBS) and incubated for 20 h at 4 °C. For analysis, mouse sera were diluted 1:100 in PBS/0.1% Tween®20 (PBST) with 1% powdered milk and allowed to react with the Gc protein for 1 h. To confirm reliability and repeatability, a blank, a negative control, and a Gc-specific mouse monoclonal antibody (11E7, dilution 1:50,000, BEI Resources, NIAID, NIH, USA) were analyzed on each plate. Detection was performed using polyclonal goat anti-mouse immunoglobulins/HRP (Agilent DAKO; P044701-2, dilution 1:1000, 30 min of incubation), 3,3’,5,5’-tetramethylbenzidine (TMB) substrate solution and TMB-Stop Solution. The optical density (OD) was determined at 450–620 nm using an automated spectrophotometer (PHOmo, Autobio Labtec Instruments Co., Ltd., Zhengzhou, China or Synergy LX, Agilent BioTek, Winooski, USA). Each control and serum was analyzed in duplicate. The cut-off for a positive antibody response was calculated as the mean OD value of the results from 25 negative mouse sera plus four standard deviations.

### CCHFV whole virus ELISA (IgG)

To prepare CCHFV antigen for the ELISA, CCHFV strain Afg09-2990, (passage +3 upon receipt, GenBank accession numbers: HM452305.1, HM452306.1, HM452307.1) was used. Supernatants of CCHFV-infected Vero C1008 (ATCC CRL-1586) cells (MOI 0.01) were collected 3 days p.i. and clarified from cell debris (10 min, 2500 rpm). Viral particles were concentrated through a sucrose cushion and purified by ultracentrifugation at 28,000 rpm for 120 min at 4 °C. Pellets were resuspended in PBS and inactivated by addition of a final concentration of 0.05% beta propiolactone (from 98.5%, pharma grade, Ferak Berlin GmbH, Berlin, Germany) for 72 h. The inactivation process has been verified. Mock antigen was prepared following the same procedure as for CCHFV antigen. Small volumes of antigen preparations were stored at −20 °C until use. High-binding single-break strip microtiter plates (Cat.No.705074, Greiner bio-one, Kremsmünster, Austria) were coated with 50 µl CCHFV or mock antigen (both diluted 1:50 in PBS) and incubated for 20 h at 4 °C. Incubation times and buffers were used as described elsewhere for an Ebola virus antigen-based ELISA^24^ Mouse sera and controls were diluted 1:100 in PBST containing 1% milk powder and allowed to react with CCHFV and mock antigen for 1 h. To confirm reliability and repeatability a blank, a negative control and a nucleoprotein-specific mouse monoclonal antibody (9D5, BEI Resources, NIAID, NIH, USA) was used on each plate. Detection was performed as described above for the Gc-specific ELISA. Each control and serum was analyzed once and the OD value of each sample on mock antigen was substracted from the OD value on CCHFV antigen to obtain corrected OD values (ΔOD). The cut-off for a positive antibody response was initially calculated as the mean OD value of the results from 25 negative mouse sera plus four standard deviations (Gc: 0.08 OD; whole virus: 0.17 OD).

### Clinical serum chemistry

Serum clinical chemistry was assessed using the Piccolo Xpress Chemistry Analyzer and the General Chemistry 13 panel (both Abaxis, Union City, USA) to determine levels of the liver enzymes aspartate aminotransferase (AST) and alanine aminotransferase (ALT) as an indicator of hepatocellular injury according to the manufacturer’s instructions.

### Statistical analysis

The statistical analysis was carried out using Graphpad Prism 10 software.

## Supplementary information

Supplementary information

## Data Availability

The datasets generated and/or analyzed during the current study are not publicly available because they are part of ongoing analyses, but are available from the corresponding author on reasonable request.
