# Supplementary material for: Side-by-side evaluation of two Crimean-Congo hemorrhagic fever virus isolates in IFNAR−/− mice
Source: Npj Viruses. 2026 Jul 31;4:35. doi: 10.1038/s44298-026-00216-2 (PMC13427833; doi:10.1038/s44298-026-00216-2)
Supplement: Supplementary file 1 — Supplementary information [file 44298_2026_216_MOESM1_ESM.pdf]

Supplementary material

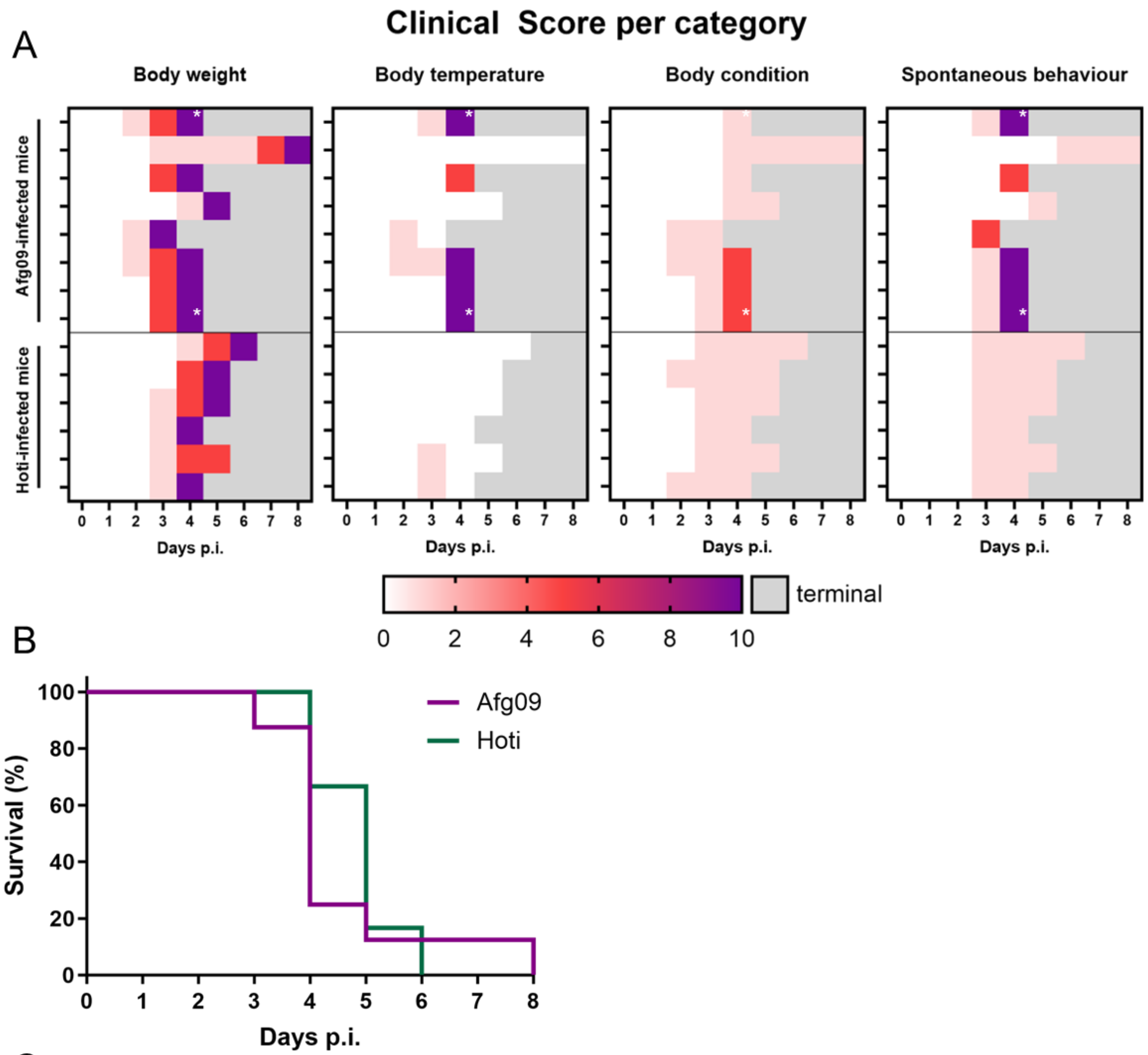

C

Scoring System	0	1	5	10
**Body weight**	0–5% reduction of the individual body weight	6–10% reduction of the individual body weight	11–15% reduction of the individual body weight	>15% reduction of the individual body weight
**Body temperature**	No deviation from physiological reference range (36.5°C-38.5°C)	Deviation of 1–1.4°C from physiological reference range	Deviation of 1.5–2.0°C from physiological reference range	Deviation of >2.0°C from physiological reference range
**Body condition**	Fur smooth, shiny; body orifices clean, pink; eyes clear, bright	Fur slightly ruffled; reduced or excessive grooming	Fur dull, disordered; unclean body orifices; eyes cloudy	Focally matted or rough fur; cloudy eyes; diarrhea; jaundice or signs of coagulopathy
**Spontaneous Behavior**	Normal behavior (sleeping, response to touch, curiosity, social interaction)	Slight deviations from normal behavior	Moderate changes in activity (hyperactivity/hypoactivity)	Severe activity changes or lethargy; self-isolation; aggressive behavior toward self or cage mates

S1 Fig. Clinical course of CCHFV Afg09 and Hoti-infected IFNAR$^{-/-}$ mice. IFNAR$^{-/-}$ mice were infected with either 100 $TCID_{50}$ CCHFV Afg09 (purple, n = 8) or Hoti (green, n = 6). **(A)** The clinical score per category was monitored daily. After reaching the clinical endpoint

(summerized score in all categories of ≥10 or ≥6 on two consecutive days), the mice were sacrificed or found dead (terminal, grey). **(B)** Survival over time (%). **(C)** Clinical Score table.

Virus	Sex	Terminal endpoint (dpi)	Deceased	Final score	Final relative body weight (%)	Maximum relative body temperature increase (°C); dpi	Final relative body temperature (°C)	Final CCHFV Genome copies Serum (copies/ng RNA, qPCR)	Final CCHFV genomes Liver (Area %, ISH)	Final CCHFV genomes Brain (Area %, ISH)	Final Gc ELISA (ΔOD)	ALT (U/l)	AST (U/l)
Afg09	M	4	x	31	-19.0	1.7; 3	-14.6	N/A	11.594	0.220	N/A	N/A	N/A
	M	8	-	12	-17.7	1.0; 6	-1.2	<LLOD	0.001	0.004	1.9	85	160
	F	4	-	21	-17.7	0.5; 3	-2.2	17,846	15.707	0.485	0.1	3,300	4,780
	F	5	-	12	-16.4	0.8; 4	-0.3	11,536	26.595	0.089	0.1	3,785	5,595
	M	3	-	16	-16.2	1.7; 3	-1.0	21,071	14.354	0.077	0.0	1,855	3,565
	M	4	-	35	-18.2	2.0; 3	-14.5	104,642	17.153	0.923	0.0	5,935	10,000
	F	4	-	35	-16.1	0.7; 3	-14.7	93,718	23.942	2.197	0.0	N/A	N/A
	F	4	x	35	-17.4	1.0; 2	-18.3	N/A	23.500	0.675	N/A	N/A	N/A
Hoti	M	6	-	12	-18.5	1.2; 4	-0.4	<LLOD	1.641	0.390	1.5	N/A	N/A
	M	5	-	12	-18.5	1.0; 3	-1.3	99	3.886	0.099	0.1	1,220	2,435
	M	5	-	12	-16.9	1.2; 3	-1.1	48	4.089	0.097	0.1	N/A	N/A
	F	4	-	12	-15.3	2.1; 3	0.5	441	3.462	0.079	0.0	1,655	3,005
	F	5	-	7	-13.5	1.7; 3	-1.1	10	1.782	0.051	0.1	N/A	N/A
	F	4	-	12	-17.1	1.9; 3	0.0	479	3.895	0.041	0.1	2,785	4,555

S2 Fig. Individual clinical course, analysis of CCHFV viral load and clinical chemistry of liver enzymes of CCHFV Afg09 and Hoti-infected IFNAR$^{-/-}$ mice. IFNAR$^{-/-}$ mice were infected with either 100 $TCID_{50}$ CCHFV Afg09 (n = 8) or Hoti (n = 6). The sex, terminal endpoint, final score, final relative body weight (%), maximum relative body temperature, final relative body temperature, final CCHFV genome copies (serum, qPCR), final CCHFV genomes (liver/brain, ISH), final serum Gc ELISA, ALT or AST are shown per individual mouse. M: male; F: female; N/A: not available; LLOD: lower limit of detection.

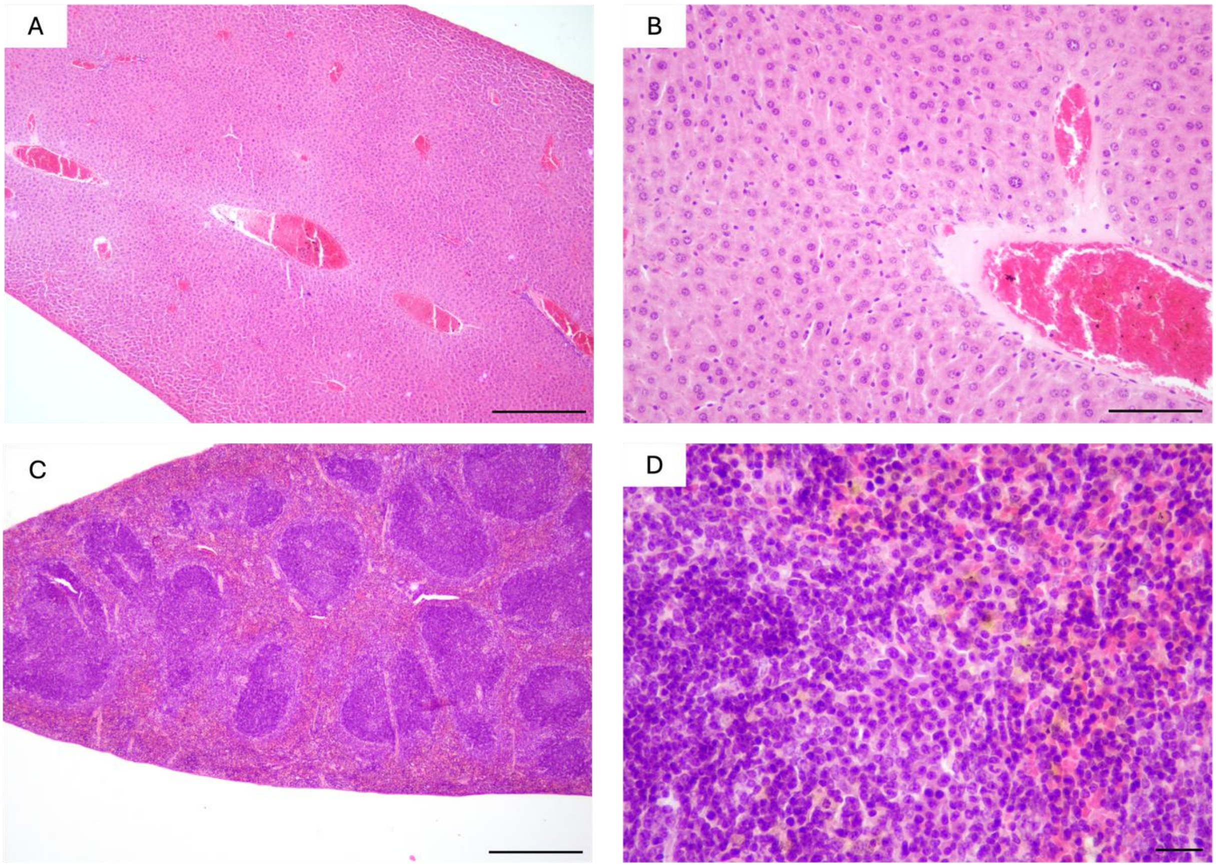

S3 Fig. Liver and spleen histopathology of untreated control mouse. (A), (B): liver; (C), (D): spleen. (A), (C): 40x magnification, (B): 200x magnification, (D): 400x magnification. No histopathological alterations were observed.
